# What the urologist needs to know before radical prostatectomy: MRI effective support to pre-surgery planning

**DOI:** 10.1007/s11547-024-01831-w

**Published:** 2024-06-25

**Authors:** Ludovica Laschena, Emanuele Messina, Rocco Simone Flammia, Antonella Borrelli, Simone Novelli, Daniela Messineo, Costantino Leonardo, Alessandro Sciarra, Antonio Ciardi, Carlo Catalano, Valeria Panebianco

**Affiliations:** 1 Department of Radiological Sciences, Oncology and Pathology, Sapienza University of Rome, Rome, Italy; 2 Department of Urology, IRCCS Regina Elena National Cancer Institute, Rome, Italy; 3 Department of Surgery, Sapienza University of Rome, Rome, Italy; 4 Department of Mechanical and Aerospace Engineering, Sapienza University of Rome, Rome, Italy; 5 Liver Failure Group, Institute for Liver and Digestive Health, UCL Medical School, Royal Free Hospital, London, UK; 6 Department of Maternal-Infant and Urological Sciences, Sapienza University of Rome, Rome, Italy

**Keywords:** Prostate cancer, Radical prostatectomy, Urinary incontinence, Erectile dysfunction, Magnetic resonance imaging, Risk-stratification

## Abstract

**Background:**

Radical prostatectomy (RP) is recommended in case of localized or locally advanced prostate cancer (PCa), but it can lead to side effects, including urinary incontinence (UI) and erectile dysfunction (ED). Magnetic resonance imaging (MRI) is recommended for PCa diagnosis and staging, but it can also improve preoperative risk-stratification.

**Purpose:**

This nonsystematic review aims to provide an overview on factors involved in RP side effects, highlighting anatomical and pathological aspects that could be included in a structured report.

**Evidence synthesis:**

Considering UI evaluation, MR can investigate membranous urethra length (MUL), prostate volume, the urethral sphincter complex, and the presence of prostate median lobe. Longer MUL measurement based on MRI is linked to a higher likelihood of achieving continence restoration. For ED assessment, MRI and diffusion tensor imaging identify the neurovascular bundle and they can aid in surgery planning. Finally, MRI can precisely describe extra-prostatic extension, prostate apex characteristics and lymph-node involvement, providing valuable preoperative information for PCa treatment.

**Conclusions:**

Anatomical principals structures involved in RP side effects can be assessed with MR. A standardized MR report detailing these structures could assist urologists in planning optimal and tailored surgical techniques, reducing complications, and improving patients’ care.

## Introduction

Prostate cancer (PCa) is a complex disease, mostly due to its multifactorial etiology, and therefore it should be promptly recognized at an early stage [1]. Indeed, when diagnosed at an advanced stage, PCa is often aggressive and it requires multimodal therapy [2]. Radical prostatectomy (RP) is recommended in case of localized or locally advanced disease, with or without extended lymph node dissection (eLND) [3]. RP procedures have progressed from perineal and retropubic open approaches to laparoscopic and robotic-assisted techniques [4].

RP-related risks are in the same associated to any other major surgery; nevertheless in some cases we can witness some side effects, more frequently urinary incontinence (UI) and erectile disfunction (ED) [5, 6]. UI etiology is multifactorial, but damages to anatomic support and pelvic innervation have been indicated as important causing factors [7]. Furthermore, despite the increasing spread of nerve-sparing approaches, the ED related to neuropraxia remains a relatively common consequence [7].

Considering the diagnostic PCa work-up, magnetic resonance imaging (MRI) is indicated by all the major international guidelines as a key tool for PCa detection and staging, being noninvasive and representing the base of prostate biopsy planning [8–11]. Prostate mpMRI should be acquired and reported using Prostate Imaging Reporting & Data System version 2.1 (PI-RADS v2.1) recommendations [12] according to the European Association of Urology (EAU) guidelines [5]. Other important advantages of performing MRI are that it can improve preoperative risk stratification and it can describe predisposing factors leading to RP side effects [13].

Therefore, the aim of this nonsystematic narrative review is to provide an overview of these factors, highlighting the anatomical and pathological aspects that should be included in a structured report. A precise and comprehensive report could significantly aid urologists on the pre-surgery planning, furnishing insightful data.

## Evidence acquisition

This nonsystematic narrative review was performed using existing literature on RP techniques, principal side effects and MR potential role in pre-surgery planning.

Online databases (Medline, PubMed, and Web of Science) were searched for original articles, systematic review and meta-analysis, expert opinion papers and international guidelines.

## Prostate anatomy

The prostate gland is a dense fibromuscular gland located in the pelvis directly below the bladder and surrounding the proximal urethra. The gland is enclosed by a fibrous capsule, with nerves and vascular bundle which is surrounded by a visceral layer of the pelvic fascia. It is composed by three anatomical zones presenting different histology: (1) the central zone constitutes the base of the gland enclosing the ejaculatory ducts; (2) the peripheral zone represents the largest zone and surrounds most of the central zone and partially the distal part of the prostatic urethra; it is the area most frequently involved by cancer; (3) the transition zone is a small glandular zone surrounding a portion of the urethra between the urinary bladder and the verumontanum. An additional anterior area can be described, and it is composed by fibromuscular stroma [14–17]. A key structure is represented by the neurovascular bundle (NVB), which is composed by vessels and neural fibers arising from the inferior hypogastric plexus (IHP) and the cavernous nerves (CN), which themselves arises from the lower portion of the IPH. The IHP, comprising fibers from both the sympathetic and parasympathetic systems, plays a crucial role in the processes of erection, ejaculation, and urinary continence; the CN are involved in the erectile function. The NVB also encompasses nerve fibers directed to structures such as the urethral sphincter. The NVB is predominantly located posterior and lateral to the prostate; only minimal nerve fibers are observed on the anterior surface of the bladder neck and the prostate. Caution is necessary during the dissection of the lateral prostatic pedicles at the prostatic base, as the NVB is closely located. There is a risk of injury to the NVB during the ligation and dissection of the pedicles or due to traction on the surrounding tissues [18, 19].

## Prostate *cancer* local staging

For tumor staging, the Tumor, Node, Metastasis (TNM) system is universally applied, reflecting intrinsic characteristics of tumor aggressiveness, and considering a schematic representation of tumor extent and pathological tumor grade (Table 1) [20]. Another widely accepted classification system is the EAU risk grouping, which is essentially based on D’Amico’s classification system. It combines clinical information on tumor extent, PSA and pathology results [5, 21] (Table 2). EAU guidelines strongly recommend using the TNM classification for PCa staging and EAU risk group stratification for prognostic subgrouping of patients. Moreover, they still assess that clinical stage should be based on digital rectal examination (DRE) only and that additional staging data furnished by imaging should be reported separately [5].

**Table 1:** Risk grouping of prostate cancer adapted from European Association of Urology [5]

Localized prostate cancer	
Low-risk	PSA < 10 ng/mL and GS < 7 (ISUP grade I) and cT1-2a^*^
Intermediate-risk	PSA 10–20 ng/mL or GS 7 (ISUP grade 2/3) or cT2b^*^
Locally advanced prostate cancer	
High-risk	- PSA > 20 ng/mL or GS > 7 (ISUP grade 4/5) or cT2c^*^ - any PSA, any GS (any ISUP grade) and cT3-4* or cN + ^§^

^*^Based on digital rectal examination

^§^*Based on CT/bone scan*

PSA, prostate specific antigen; GS, Gleeson Score; ISUP, International Society of Urological Pathology

**Table 2:** Prostate cancer Tumor, Node, Metastasis staging system

T-stage	
Tx T0	Primary tumor cannot be assessed No evidence of primary tumor
T1 T1a T1b T1c	Clinically inapparent tumor not palpable nor visible by imaging Incidental tumor ≤ 5% of tissue Incidental tumor > 5% of tissue Tumor detected by needle biopsy
T2 T2a T2b T2c	Organ confined and palpable tumor One half of one lobe involved More than half of one lobe (not both) Both lobes
T3 T3a T3b	Palpable extension through prostate capsule Extracapsular extension (uni- or bilateral) Invasion of seminal vesicles
T4	Tumor invades external sphincter, rectum, levetor muscle, pelvic side wall
N Stage	
Nx N0	Regional nodes cannot assessed No regional lymph nodes (below level of bifurcation of common iliac arteries)
N1	Regional node metastases – including pelvic, hypogastric, obturator, iliac, sacral
M stage	
M0	No distant metastasis
M1 M1a M1b M1c	Distant metastasis Non regional lymph nodes Bone Other site(s)

## Surgical treatment

### Radical prostatectomy

Radical prostatectomy (RP) is recommended in case of localized or locally advanced disease, and it can be performed with open, laparoscopic, and robot-assisted approaches. The open RP can be performed via perineal or retropubic approaches, even if this technique is now obsolete. The robot-assisted technique introduced the combination of minimally invasive advantages of laparoscopic RP with improved surgeon ergonomics and greater technical ease of the reconstruction of the vesicourethral anastomosis [5].

Currently, the main surgical approach in use is the nerve-sparing technique, which can maintain the integrity of the NVB. This approach is envisioned considering the distance between the NVB and the foci of prostate cancer in the specimen evaluated during the procedure [22]. Indeed, to date there is no consensus on standardized clinical and radiological information that could set specific indications in this setting. Nerve sparing technique is crucial for postoperative side effects, but is correlated with an higher risk of positive surgical margins [23].

A novel robotic technique is represented by Retzius-Sparing Robot-Assisted Laparoscopic Radical Prostatectomy (RS-RARP), which spares the retropubic space by passing through the pouch of Douglas avoiding bladder mobilization and preserving the endopelvic fascia, the Santorini’s plexus and the puboprostatic ligaments, drastically reducing urinary incontinence onset. There are various surgical approaches used in RARP and these techniques primarily depend on the surgical anatomy of the periprostatic fascias. The connection between the lateral pelvic fascia and the prostate capsule determines the localization of NVB. The intrafascial plane refers to the space included between the prostate capsule and the prostatic fascia. The interfascial plane is the space between the prostatic fascia and the lateral pelvic fascia. Before initiating dissection in the intrafascial or interfascial planes, the endopelvic fascia must be incised along the tendinous arch of the pelvic fascia. The extrafascial plane involves the outer part of the NVB and is a non-nerve-sparing technique. Therefore, a nerve sparing technique can be achieved through either interfascial or intrafascial dissection [23]. The Pasadena consensus panel proposed a different terminology for the dissection planes to clarify the level of nerve preservation and it helps to differentiate and describe the extent of nerve preservation in each respective dissection plane. They suggested using the terms full nerve-sparing for intrafascial dissection, partial nerve-sparing for interfascial dissection, and minimal nerve-sparing for subextrafascial [24].

### Radical prostatectomy side effects

Urinary incontinence (UI) is one of the most common complications after RP. It has a complex etiology that can be caused by bladder or sphincter dysfunction, or a combination of the two. Although pre-existing bladder changes may affect UI after RP, sphincter dysfunction caused by tissue injury associated with prostate dissection is thought to be the most significant cause. Moreover, RP reduces the length and maximal closure pressure of the urethra. Another common side effect after RP is erectile dysfunction (ED) (still 10–46% cases), albeit with a decreasing trend of its frequency thanks to the increasing use of the nerve sparing approaches [25].

## Prostate MRI assessment

### PI-RADS score and Image quality

Prostate MRI should be acquired and reported according to the recommendations from PI-RADS v2.1, a scoring system designed to improve the detection, localization, characterization, and risk stratification in patients with suspected PCa [5, 12, 26]. However, prostate MRI, being technically demanding, must be acquired with high image quality standards, in order to reach its full diagnostic power [27, 28]. With the introduction of PI-QUAL score (Prostate Imaging Quality) a new standardized assessment of MRI scans quality has been achieved [29].

According to PI-RADS v2.1 the acquisition protocol includes T2-weighted imaging (T2WI) on the axial plane and on at least an orthogonal plane (coronal or sagittal plane), diffusion-weighted imaging (DWI) obtained with low b-value (preferentially 50–100 s/mm2), intermediate ones (800–1000 s/mm2) and high one (≥ 1400 s/mm2), with apparent diffusion coefficient (ADC) map reconstructions, built on at least 3 b values < 1000. Dynamic-contrast enhanced (DCE) images after intravenous contrast injection should be acquired too (Table 3) [12, 30, 31].

**Table 3:** Parameters of the magnetic resonance imaging protocol according to PI-RADS v2.1 recommendations, using both 3 T and 1.5 T scanners

Sequence		
T2WI	FOV (cm)	12–20
	Slice thickness (mm)	3
	In plane dimension (mm x mm)	≤ 0.7 x ≤ 0.4
DWI	FOV (cm)	16–22
	Slice thickness (mm)	≤ 4
	TE (msec)	≤ 90
	TR (msec)	≥ 3000
	In plane dimension (mm)	≤ 2.5
	b-value (sec/mm2)	0–100; 800–100; ≥ 1400
DCE	FOV	Including both the entire prostate and seminal vesicles
	Temporal resolution (s)	< 15
	Slice thickness (mm)	3
	TE (msec)	< 5
	TR (msec)	< 100
	In plane dimension (mm x mm)	≤ 2 x ≤ 2
	Temporal resolution (s)	≤ 15

PI-RADS, Prostate Imaging Reporting and Data System; T2WI, T2-Weghited Images; FOV, Field of View; DWI, Diffusion-Weighted Imaging; TE, Time of Echo; TR, Time of Repetition; DCE, Dynamic Contrast-Enhanced

### MRI techniques not included in the PI-RADS system

Diffusion tensor imaging (DTI) and tractography have evolved as noninvasive imaging techniques that provide in vivo information on fiber anatomy by providing a three-dimension (3D) visualization. The method is based on the sensitivity of water protons observed in the microstructural environment [32, 33]. According to fiber myelination, axonal membrane, and sub-voxel coherence, the diffusion pathways of water molecules follow the longitudinal axis of myelinated fibers rather than the perpendicular one. Furthermore, direction-dependent diffusion may be inferred by using diffusion sensitizing gradients in distinct directions [33, 34]. This data is represented in diffusion tensor maps, which furnish insights into the underlying tissue's microstructure and properties. Therefore, the macroscopic diffusion data may be used to elicit qualitative changes in fiber diameter and extracellular fraction, while rigorous diffusion modeling can yield quantitative values for these parameters. In addition to information on microstructure analyzed, DT can be used to evaluate tissue architecture, such as fiber length and density [32]. This technology can be used to map periprostatic nerve fibers, to recognize the entire neurovascular bundle and to locate it, giving important information before RP. DTI can be used also to detect sphincters and membranous urethra fibers, which present circular and longitudinal fibers, respectively. Indeed, thanks to quantitative DTI it is feasible to differentiate this kind of different fibers [8, 32, 35]. However, DTI is strongly influenced by image quality, suffering artifacts [33, 36].

## What to look for on MR images before surgery to avoid complications

Description and measurements of anatomical features on preoperative prostate MRI are employed in risk models for treatment decisions to predict predisposing factors of the most common side effects and minimize positive RP margins [37]. Most of these adverse effects are debilitating and affect the quality of life of patients. Therefore, it is crucial to identify in MR images the structures involved in generating process of these side effects, to prevent or reduce their severity.

### Structures involved in urinary incontinence

**Fig. 1:**
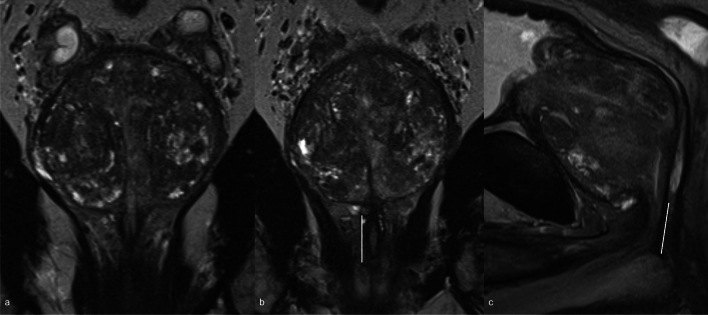
T2WI on coronal plane (**a**, **b**) and sagittal plane (**c**) where is evident the MUL. The white line indicates the correct measurement of MUL, showing how the sagittal plane is more appropriate. T2WI, T2 weighted imaging; MUL, membranous urethral length

Urethra should be assessed on MRI, evaluating its length (LP) and width (WP). Prostate volume (PV) should also be calculated, using the formula for calculating the volume of an ellipsoid [38]. Other findings to be considered are membranous urethral length (MUL), urethral wall thickness (UWT), elevator ani muscle (LAM) and obturator internus muscle (OIM) thickness, ratio of elevator ani muscle/prostate volume (LAM/PV), urethra maximal diameter, and prostate-urethral angle. In particular, MUL and PV proved to be involved in UI [7, 39–41]. MUL is typically measured on sagittal or coronal T2-weighted images [42] and recent meta-analyses indicate that a longer MUL measurement based on MRI [37, 43] is linked to a higher likelihood of achieving continence restoration [43]. Moreover, MUL assessment may also be useful in identifying the best candidates for preoperative pelvic floor muscle workouts, which have been found to enhance early postoperative continence [44]. Indeed, the pre-surgery measurement of the MUL on MRI images proved to be an independent predictor of urinary continence after prostatectomy. [45]. Boellaard et al. [46] proposed new standardized recommendations to correctly measure MUL on MRI. More specifically, they suggest evaluating it in sagittal T2WI since coronal images typically lack parallel angulation to the MU and to identify the hyperintense urethral lumen and dorsal hypointense membranous structure on one of the midsagittal images. The measurement line should be placed dorsally and parallel to the hyperintense urethral lumen. The upper limit should be the prostate apex, defined as the lowest portion of the peripheral zone of the dorsal prostate; the lower limit should be considered the intersection of the urethra with the bulb of the corpus spongiosum as the landmark. Therefore, MRI MUL measurement should be included in a pre-treatment predictive model alongside other major indicators of UI, such as patient age, BMI, comorbidities profile, preoperative urinary and erectile dysfunction (Fig. 1).

Another important structure involved in UI is the urethral sphincter complex. The urethral sphincter complex comprises a cylindrical structure that encircles the urethra, extending vertically from the bladder neck to the perineal membrane. It is composed by the proximal lissosphincter, a smooth muscle and the distal rhabdosphincter, a skeletal muscle [47]. The urethral striated sphincter forms a covering over the anteriolateral urethra, resembling a hat from the bladder neck to the verumontanum. It transitions into a ring shape around the urethra from the verumontanum to the perineal membrane, culminating at the central tendon of the perineum. Moving from the bladder neck to the perineal membrane, the thickness of the urethral smooth sphincter gradually decreases, extending forward to encircle the urethra alongside the urethral striated sphincter in a ring-like fashion [47, 48]. In particular the lissosphincter muscle has been indicated as a critical structure for continence maintenance [32]. MRI enables the identification of sphincter complex as hypointense structure on T2W images. Because the muscle bundles of the posterior urethral sphincter exhibit low signal intensity on T2W images, they sharply contrast with the high signal intensity of the urethral mucosa, submucosa, and surrounding fat-containing tissues, thereby allowing for clear visualization of their contour, starting, and ending points [48]. In addiction DTI allow to detect urethral sphincter complex fibers, by providing 3D visualization of the urethral sphincter complex fibers in terms of size, location, direction, and integrity [35].

**Fig. 2:**
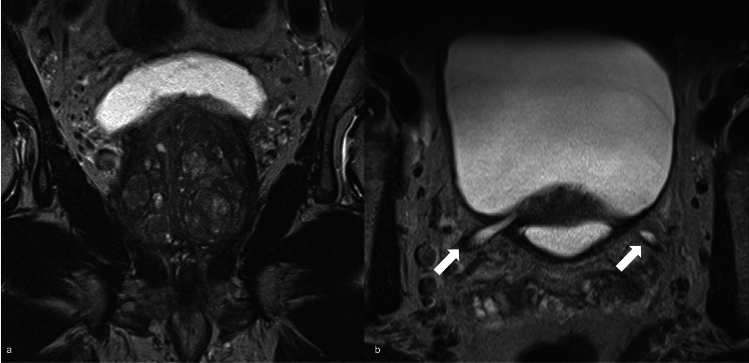
T2 weighted imaging on coronal (**a**) and axial (**b**) plane in which is evident a median lobe obstructing both ureteral ostiums, hindering their identification; consequent distal ureteral distension is detected (white arrows)

Finally, another important MRI finding to be detected and described is the presence of a prostate median lobe: the distortion of bladder neck during RP can be due to a protruded median lobe. A large median lobe can lead to an obstruction that impairs the smooth flow of urine at the bladder neck and alters the normal angle between prostate and urethra, causing abnormal bladder contractions during urination. The median lobe makes difficult to clearly see the posterior border of the prostate and bladder neck and can hinder the identification of ureters ostiums (Fig. 2). In case of bladder neck resection, the risk of injuring the ureteral orifices due to their proximity to the edge of the bladder neck becomes higher [49].

### Structures involved in erectile dysfunction

**Fig. 3:**
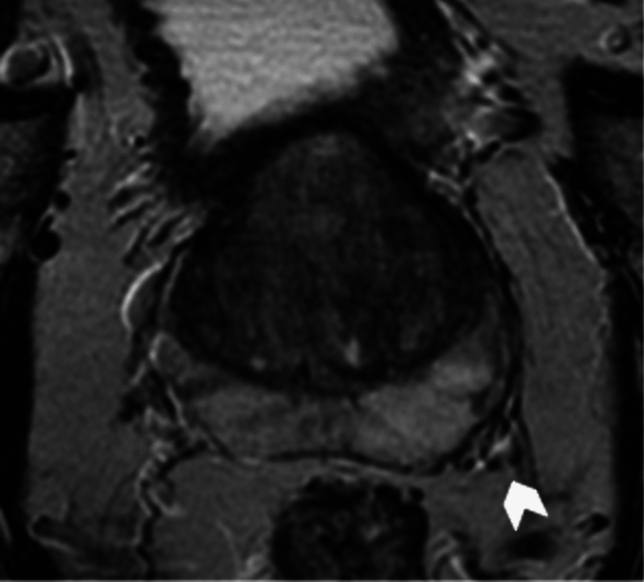
Example of T2 weighted imaging on axial plane where is visible the neurovascular bundle (white arrow head)

**Fig. 4:**
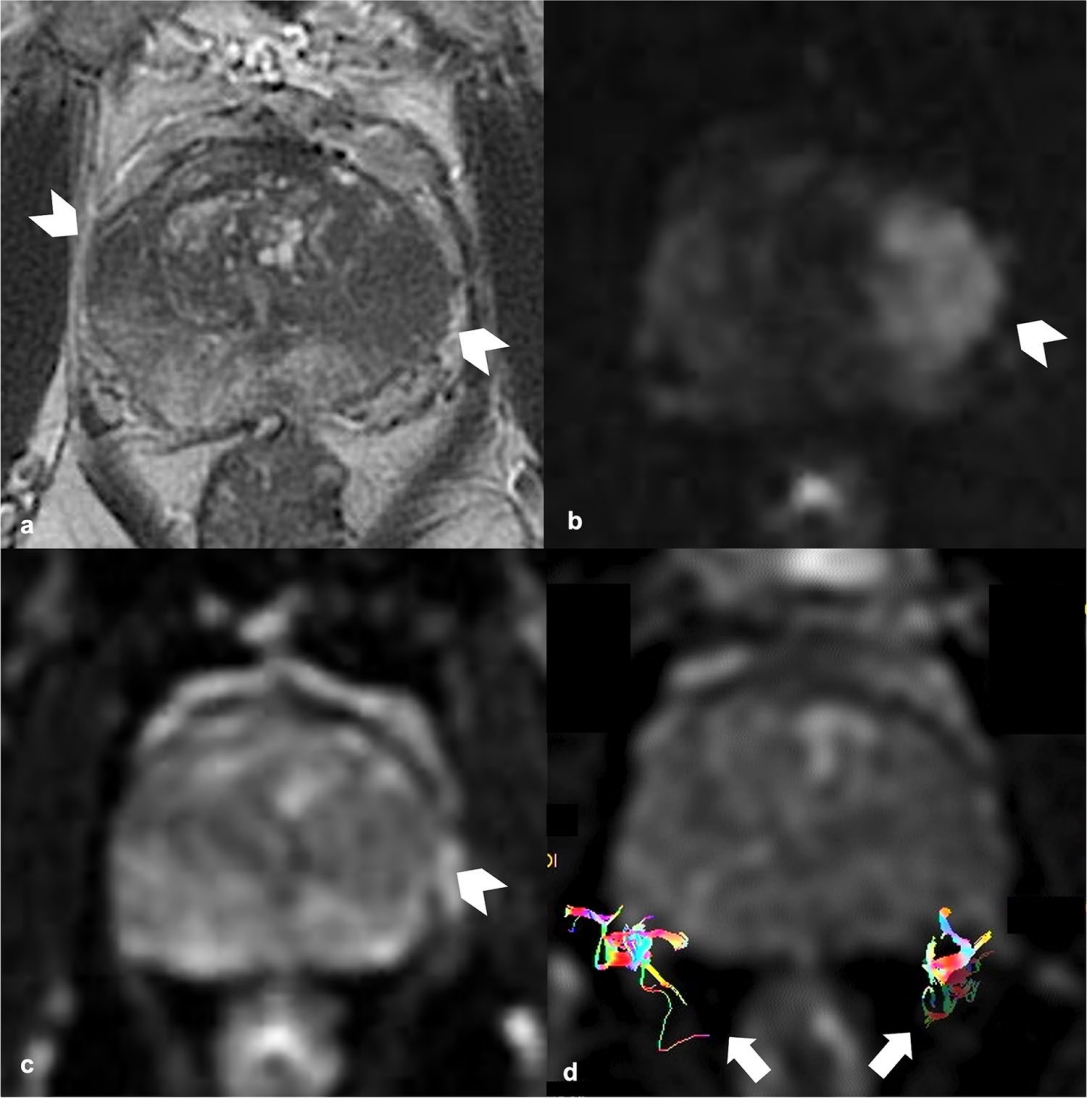
**a** T2WI (axial) shows two alterations respectively on the left lateral mid peripheral zone (white arrow head), which determines capsular bulging and on the right mid antero-lateral peripheral zone (white arrow head); **b**, **c** DWI (b = 2000) and ADC map show a focal and marked hyperintensity on high b-value and focal hypointensity on ADC map on the left lateral peripheral zone lesion (white arrow head), with capsular bulging. **d** DTI reveals that the lesion is distant from the NVB (white arrows). T2WI, T2 weighted imaging; DWI, Diffusion Weighted Imaging; ADC map, Apparent Diffusion Coefficient map; DTI, Diffusion Tensor Imaging; NVB, neurovascular bundle

The neurovascular bundle (NVB) crosses extremely close to the prostate gland and carries blood flow as well as sympathetic and parasympathetic neural branches to the corpora cavernosa and the external urinary sphincter muscle, playing a key role in penile erection and urine continence. Approximately 52% of nerves are distributed along the lateral surface of the prostate, while the remaining 48% forms a distinct bundle in the posterior-lateral region [50]. One possible side effect during RP is the neurapraxia, due to the traction of periprostatic neurovascular fibers, during the dissection of the gland. The drafting of standardize nomograms to predict ED after RP have been largely proposed, without significant results [22, 51]. The NVB has low signal intensity on T1-and T2-WI and it is surrounded by a fat plane with characteristic high signal intensity [52] (Fig. 3). MRI and DTI could be used to locate the NVB and periprostatic neurovascular fibers (PNF) to furnish key pre-treatment information for surgery planning, to prevent UI and ED (Fig. 4).

### Extra-prostatic extension and surgical margins

**Fig. 5:**
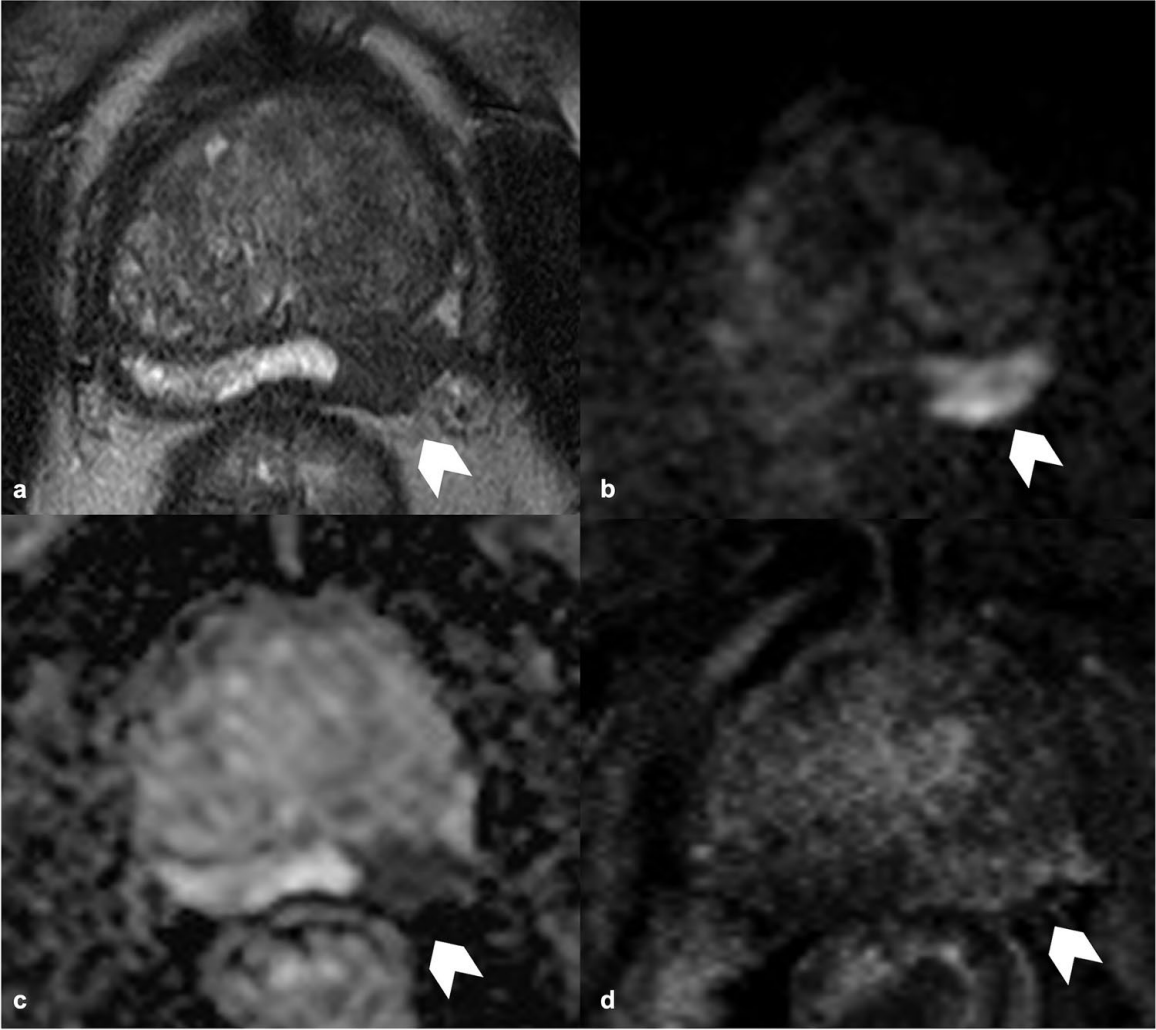
**a** T2WI on axial plane shows an alteration on the mid left posterior-lateral peripheral zone (white arrow head); **b**, **c** DWI (b = 2000) and ADC map show a focal and marked  hyperintensity on high b value and hypointensity on ADC map on the left peripheral zone lesion (white arrow head); **d** DCE shows an early and focal enhancement of the lesion. All the sequences demonstrate a lesion contact with the capsule measuring about 15 mm. T2WI, T2 weighted imaging; DWI, Diffusion Weighted Imaging; ADC, Apparent Diffusion Coefficient; DCE, dynamic contrast enhancement

**Fig. 6:**
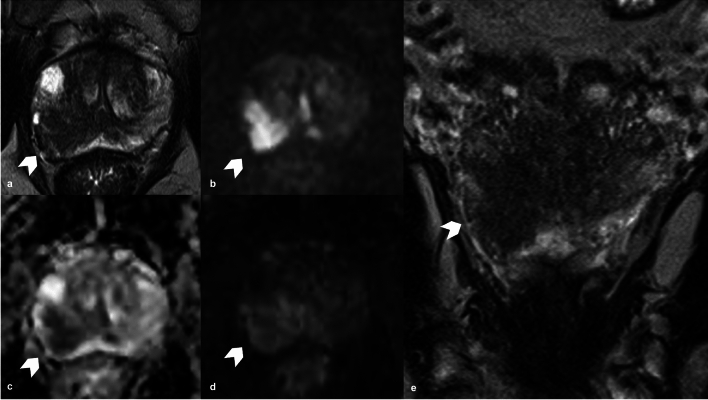
**a**, **e** T2WI (axial and coronal plane) show an alteration on the mid right posterior-lateral peripheral zone (white arrow head), distant from the NVB; **b**, **c** DWI (b = 2000) and ADC map show a focal and marked hyperintensity on high b value and hypointensity on ADC map on the right posterior lateral peripheral zone lesion (white arrow head); **d** DCE shows an early and focal enhancement of the lesion. All the sequences demonstrate marked capsular bulging. T2WI, T2 weighted imaging; DWI, Diffusion Weighted Imaging; ADC map, Apparent Diffusion Coefficient; NVB, neurovascular bundle; DCE, dynamic contrast enhancement

**Fig. 7:**
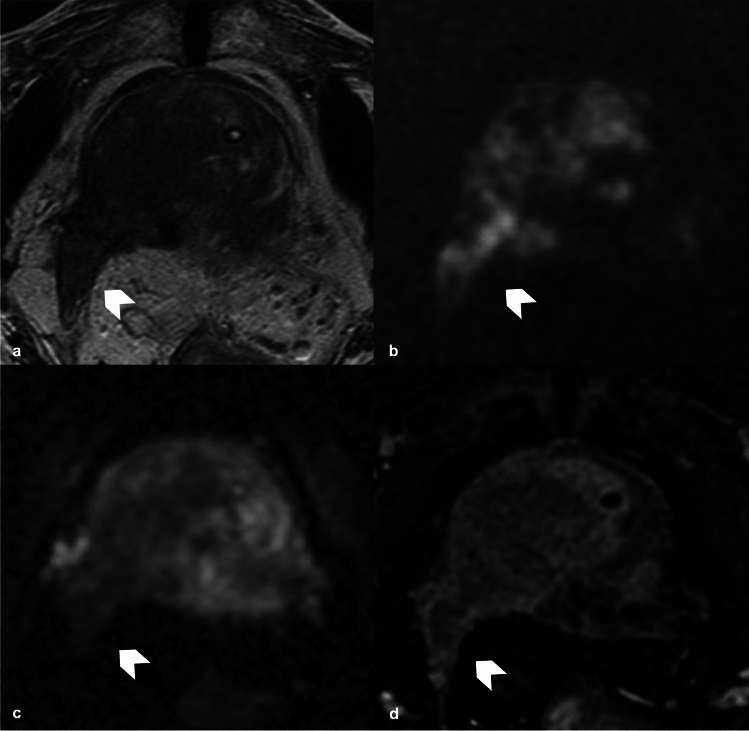
**a** T2WI (axial plane) shows an alteration on the right mid-base peripheral and transitional zone (white arrow head); **b**, **c** DWI (b = 2000) and ADC map show a focal and marked hyperintensity on high b value and hypointensity on ADC map on the right mid-base zone (white arrow head); **d** DCE shows early and focal enhancement of the lesion. All the sequences demonstrate the infiltration of the right seminal vesicle. T2WI, T2 weighted imaging; DWI, Diffusion Weighted Imaging; ADC map, Apparent Diffusion Coefficient; DCE, dynamic contrast enhancement

To date MRI is used to detect and localize suspected prostate cancer and also to guide biopsies [53, 54]. However, MRI has a potential role for local preoperative staging and the identification of extra-prostatic extension and the infiltration of the nearby anatomical structures (i.e., bladder neck and rectum), features that carries the patient to an higher stage and to a worse prognosis, due to an higher risk of positive margins after RP and therefore a greater incidence of recurrence [55, 56]. Indeed, extra-prostatic extension (EPE) (T3a and T3b) in PCa is linked to an increased risk of biochemical recurrence and metastatic disease after RP or radiation therapy [57]. Several MRI features are associated with pathological EPE such as tumor size and volume, the length of tumor contact with the capsule (Fig. 5), capsular tumor’s bulging (Fig. 6), fat tissue infiltration and seminal vesicles invasion (Fig. 7), that could be easily identified on a T2W imaging and on functional imaging (DWI and DCE) [54, 58]. In particular, a greater length of PCa contact with the capsule on histopathology correlates with higher probability of EPE; therefore, it represents an independent predictor for EPE detection. Prostate MRI demonstrated moderate sensitivity and high specificity to detect extra-capsular extension, seminal vesicle involvement or T3 stage [13]. The definition of seminal vesicles involvement is essential, and Martini et al. [59] proposed a new nomogram, including MRI evaluation of seminal vesicles infiltration, with promising results in the setting of risk-stratification of patients who can be directed to nerve sparing RP.

### Prostate apex description

Achieving sufficient urethral length after RP can be challenging in case of prostate apical prominence [60]. Indeed, Lee et al. [60] proposed a new classification of prostatic apex shape on MR images: type A defined as a prostatic apex overlapping the membranous urethra both anteriorly and posteriorly; type B and C only anteriorly or posteriorly, respectively; type D with no signs of overlapping. The last group proved to be linked to an higher rate of early return to urinary continence. Furthermore, a more recent experience too proved how type C and D were associated to very early continence restoration after catheter removal following RP [61]. Moreover, the prostate apex can be close to the skeletal fibers of the urogenital diaphragm, and therefore apical lesions should be precisely described because they might involve these structures. Indeed, in prostatectomy specimens, positive apical margins can be found, with cancerous cells reaching the skeletal muscle [62].

### Lymph-nodes involvement

For patients with non-metastatic PCa undergoing RP, European Association of Urology guidelines [5] suggest considering an ePLND for high- and intermediate-risk cases exploiting validated nomograms, such as the Briganti [63], the Gandaglia [64] or the Memorial Sloan Kettering Cancer Center [65] nomograms. However, imaging, and more specifically MRI, proved to play an important role in nodal assessment and Node-RADS score has been proposed [66]. It can be applied to any anatomical site, aiming to standardize the reporting, exploiting dimension and configuration criteria to assign a 5-point category score, to define the likelihood of nodal involvement. Node-RADS score applied to the specific pre-RP evaluation proved a very high specificity, suggesting its potential role in addition to the already mentioned nomograms [3].

## Discussion

**Fig. 8:**
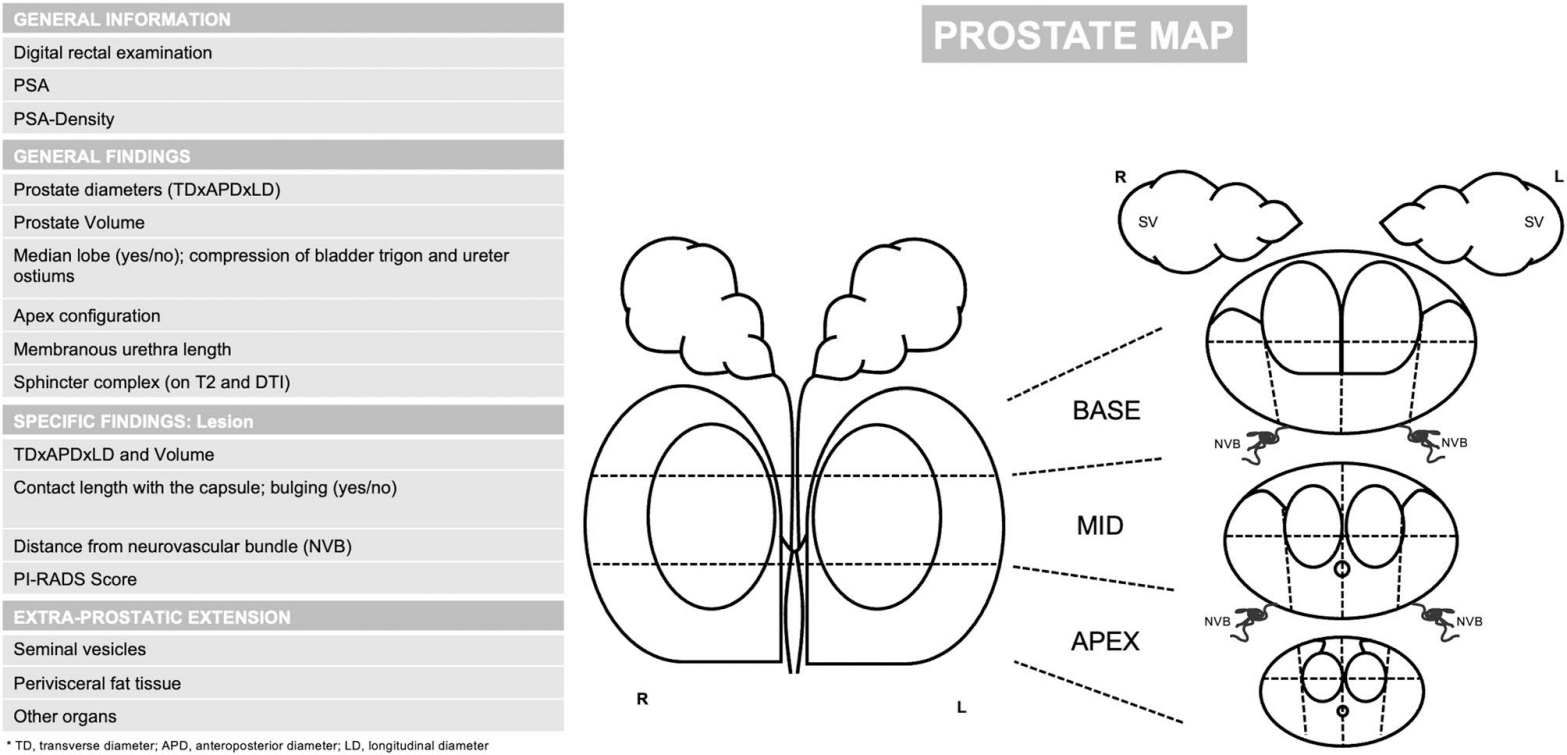
Example of a pre-radical prostatectomy magnetic resonance imaging structured report

Since prostate cancer is a highly incident disease, with an increasing number of possible surgical approaches, it is necessary to standardize preoperative assessment to minimize long-term side effect and consequently improve patients’ quality of life. When reading an MRI, especially if positive for PCa, it is mandatory to precisely describe all the anatomical structures involved in possible RP side effects and all the extra-prostatic structures eventually infiltrated. It would be important to describe all these elements in structured radiological reports (Fig. 8), to precisely furnish all the information that are useful for the pre-surgery planning. This standardized MR approach could help urologists to have a clearer view of the local stage of the neoplasm, to consequently choose the most appropriate surgical technique.

Complications occur in 10–25% of patients who undergo RP; age, pre-treatment PSA value, patient comorbidities, body mass index, and surgeon experience are independent clinical risk factors for side effects [42]. When compared to open prostatectomy, robot-assisted approach has the benefits of a minimally invasive procedure, with less intraoperative blood loss, less post-surgery pain, and a shorter hospitalization time. Prostate size is associated to greater complexity of surgical procedures [42]. Mazzone et al. proposed an updated super-extended RARP technique, which is suitable for patients with posterior iT3a or iT3b as indicated in preoperative MRI [67]. This technique proves to be effective in managing locally advanced stage cases, showing favorable outcomes in terms of reduced complications and successful recovery of urinary continence [67]. Additionally, this technique may offer a promising possibility of achieving biochemical disease control and potentially delaying disease progression for low-grade tumors and negative surgical margins at the final pathology [67].

Understanding pelvic anatomy is critical for protecting functioning structures affecting continence recovery during surgery. The integrity of the distal sphincteric unit determines bladder control following RP. Sphincter muscle preparation, rhabdosphincter restoration, and sphincter-preserving anastomotic procedures are all key elements for maintaining functioning tissue. Therefore a correct radiological pre-surgical identification of this anatomical structures is crucial to prevent UI [42].

It became evident that MRI, with possible acquisition of additional DTI technique, improves the accuracy of the surgical planning also with regard to the appropriateness of preserving or resecting NVBs and sphincter fibers complex [11, 68]. Therefore the use of validated preoperative tools, including MRI features, for predicting EPE of prostate tumor could assist the urologist to correctly stratify patients for surgical planning and determine a reduction of adverse outcomes [69, 70]. Pesapane et al. reported [71] that a staging MRI should be used to either rule out EPE (when the cancer does not touch the prostatic capsule) or to predict the likelihood of EPE. Nomograms using preoperative PSA, clinical stage, and biopsy Gleason score (i.e., Partin tables) can already be used to extract such information. These provide the likelihood of EPE but do not provide other essential information, such as its amount and precise location. Both can be provided by MRI staging, either as a stand-alone test or in conjunction with PSA and/or other clinical variables. In the literature, nomograms which include MRI have been developed for these purposes; Soeterik T. et al. proposed three models outperforming nomogram without MRI as staging tool [72]. Furthemore, Martini et al. [73] developed a practical nomogram that could have broader applicability in daily medical practice to predict the risk of EPE, which has been validated too [74]. This nomogram incorporates various factors, including serum PSA level, highest ISUP grade, and the highest percentage of tumor involvement in biopsy cores, in addition to MRI findings [74]. Russo et al. [75], following the local staging system proposed by Wheeler, suggested a pre-surgical staging system based on mp-MRI (radiological Wheeler), resulting in a more precise surgical technique, selecting the degree of nerve sparing approach (Table 4). The accuracy of the proposed staging system resulted to be high, therefore mpMRI is a critical investigating tool for preoperative local staging. The key role of MRI for preoperative assessment was endorsed by Schiavina et al. too, affirming that the incidence of positive surgical margins was considerably lower in patients with a preoperative mpMRI compared to those without preoperative mpMRI (respectively 12.4% vs. 24.1%) [50].

**Table 4:** Radiological Wheeler classification of prostate cancer using magnetic resonance imaging

Organ-confined	
Radiological Wheeler Class	Description
*L0*	Tumor does not touch the capsule
*L1*	Tumor present minimal contact with the capsule without capsule alteration
*L2*	Broad contact between tumor and the capsule and/or bulging and/or inhomogeneity of capsule

Non organ-confined	
Radiological Wheeler Class	Description
*L3*	Tumor invades peri-visceral adipose tissue or balder neck smooth muscle
*L3 focal*	Slight irregularity of margin of the capsule
*L3 extended*	Discontinuity of capsule with periprostatic adipose tissue inhomogeneity or seminal vesicles infiltration

## Limitations

Unfortunately MRI criteria already in use are mostly qualitative, and still associated with an high degree of reader variability, even if PI-RADS v2.1 scoring system achieve an higher reproducibility in this setting too [54].

Moreover, even if MRI has become the gold standard for the diagnosis of PCa, it has some limitations. DWI, ADC map and DCE sequences are strongly susceptible to motion artifact, which would lead to low quality images [76]. The quality is also influenced by the presence of foreign metallic bodies (such as hip prothesis) or intrinsic patient characteristics (such as severe obesity). In this case the thickness of the adipose tissue determines an increase distance between the coil and the prostate gland, resulting in an images' deterioration, with a low-quality exams [76].

## Conclusions

MRI is recommended as first line test for prostate cancer early detection, but it could also significantly aid urologists in their preoperative planning. With this goal, MRI readers should describe in a standardized scheme all the anatomical structures pertaining the prostate gland and prostatic bed, to furnish to surgeons essential information that would lead to a precise pre-radical prostatectomy planning, with consequent reduction of side effects.
