# Correction: What the urologist needs to know before radical prostatectomy: MRI effective support to pre‑surgery planning

**DOI:** 10.1007/s11547-024-01887-8

**Published:** 2024-09-06

**Authors:** Ludovica Laschena, Emanuele Messina, Rocco Simone Flammia, Antonella Borrelli, Simone Novelli, Daniela Messineo, Costantino Leonardo, Alessandro Sciarra, Antonio Ciardi, Carlo Catalano, Valeria Panebianco

**Affiliations:** 1 Department of Radiological Sciences, Oncology and Pathology, Sapienza University of Rome, Rome, Italy; 2 Department of Urology, IRCCS Regina Elena National Cancer Institute, Rome, Italy; 3 Department of Surgery, Sapienza University of Rome, Rome, Italy; 4 Department of Mechanical and Aerospace Engineering, Sapienza University of Rome, Rome, Italy; 5 Liver Failure Group, Institute for Liver and Digestive Health, UCL Medical School, Royal Free Hospital, London, UK; 6 Department of Maternal-Infant and Urological Sciences, Sapienza University of Rome, Rome, Italy

**Correction: La radiologia medica (2024) 129:1048–1061** 10.1007/s11547-024-01831-w

In the original publication of the article, the affiliations of authors, Costantino Leonardo and Valeria Panebianco and were published incorrectly as:

**Costantino Leonardo**: Uro-Oncology Unit, IFO IRCSS “Regina Elena” National Cancer Center Institute, Via Fermo Ognibene 23, 00144 Rome, Italy.

**Valeria Panebianco**: Department of Radiological Sciences, Oncology and Pathology, Sapienza University/Policlinico Umberto I, Viale del Policlinico 155, 00185 Rome, Italy and Department of Radiological Sciences, Oncology and Pathology, Sapienza University of Rome, Viale Regina Elena 324, 00161 Rome, Italy.

However, the correct affiliations should read as follows:

**Costantino Leonardo**: Department of Urology, IRCCS Regina Elena National Cancer Institute, Rome, Italy.

**Valeria Panebianco**: Department of Radiological Sciences, Oncology and Pathology, Sapienza University of Rome, Rome, Italy.

The original article has been corrected.

